# The mobilome of *Lactobacillus crispatus* M247 includes two novel genetic elements: Tn*7088* coding for a putative bacteriocin and the siphovirus prophage ΦM247

**DOI:** 10.1099/mgen.0.001150

**Published:** 2023-12-12

**Authors:** Lorenzo Colombini, Francesco Santoro, Mariana Tirziu, Elisa Lazzeri, Lorenzo Morelli, Gianni Pozzi, Francesco Iannelli

**Affiliations:** ^1^​ Laboratory of Molecular Microbiology and Biotechnology, Department of Medical Biotechnologies, University of Siena, 53100 Siena, Italy; ^2^​ Università Cattolica del Sacro Cuore, Department of Food Science and Technologies for a Sustainable Agri-food Supply Chain (DiSTAS), University of Piacenza, 53100 Piacenza, Italy

**Keywords:** bacteriocins, complete genome, CRISPRs, insertion sequences (ISs), integrative and mobilization elements (IMEs), *Lactobacillus crispatus*, M247, mobile genetic element (MGEs), mobilome, nanopore sequencing, probiotic, prophage, siphovirus, Tn*7088*, ΦM247

## Abstract

*
Lactobacillus crispatus* is a member of the vaginal and gastrointestinal human microbiota. Here we determined the complete genome sequence of the probiotic strain M247 combining Nanopore and Illumina technologies. The M247 genome is organized in one circular chromosome of 2 336 109 bp, with a GC content of 37.04 % and 2303 ORFs, of which 1962 could be annotated. Analysis of the M247 mobilome, which accounts for 14 % of the whole genome, revealed the presence of: (i) Tn*7088*, a novel 14 105 bp long integrative and mobilizable element (IME) containing 16 ORFs; (ii) ΦM247, a novel 42 510 bp long siphovirus prophage containing 52 ORFs; (iii) three clustered regularly interspaced short palindromic repeats (CRISPRs); and (iv) 226 insertion sequences (ISs) belonging to 14 different families. Tn*7088* has a modular organization including a mobilization module encoding FtsK homologous proteins and a relaxase, an integration/excision module coding for an integrase and an excisionase, and an adaptation module coding for a class I bacteriocin and homologous to the listeriolysin S (*lls*) locus of *Listeria monocytogenes *. Genome-wide homology search analysis showed the presence of Tn*7088*-like elements in 12 out of 23 *L *. *crispatus* complete public genomes. Mobilization and integration/excision modules are essentially conserved, while the adaptation module is variable since it is the target site for the integration of different ISs. Prophage ΦM247 contains genes for phage structural proteins, DNA replication and packaging, lysogenic and lytic cycles. ΦM247-like prophages are present in seven *L *. *crispatus* complete genomes, with sequence variability mainly due to the integration of ISs. PCR and sequencing showed that the Tn*7088* IME excises from the M247 chromosome producing a circular form at a concentration of 4.32×10^−5^ copies per chromosome, and reconstitution of the Tn*7088* chromosomal target site occurred at 6.65×10^−4^ copies per chromosome. The ΦM247 prophage produces an excised form and a reconstituted target site at a level of 3.90×10^−5^ and 2.48×10^−5^ copies per chromosome, respectively. This study identified two novel genetic elements in *L. crispatus *. Tn*7088* represents the first example of an IME carrying a biosynthetic gene cluster for a class I bacteriocin in *L. crispatus *.

## Data Summary

The complete genome sequence of *Lactobacillus crispatus* M247 is available under GenBank accession no. CP088015, whereas Nanopore and Illumina sequencing reads are available under Sequence Read Archive (SRA) accession no. SRR17479173 and SRR17479172, respectively.

**Impact Statement**

Despite a huge number of bacterial genomic sequences being available in public databases, only a few are complete genomes. Furthermore, the bacterial mobilome, which can constitute up to 25 % of the whole genome, remains poorly annotated and characterized. In this work, we determined the complete genome sequence of the probiotic strain *Lactobacillus crispatus* M247 and analysed its mobilome, revealing the presence of two novel genetic elements: the integrative and mobilizable element Tn*7088* carrying an adaptation module homologous to the listeriolysin S locus of *Listeria monocytogenes *, and the siphovirus prophage ΦM247. We also demonstrated that both elements are able to produce excised forms and reconstitute the target sites in *L. crispatus* chromosomes. These findings suggest that both elements have the potential to horizontally transfer among different *L. crispatus* strains and possibly to other bacterial species of the vaginal and gastrointestinal human microbiota. These data will contribute to the understanding of the evolution of this important microorganism.

## Introduction

*Lactobacillus crispatus* is a member of the vaginal and gastrointestinal human microbiota. In healthy women, it is the most commonly isolated species among the vaginal lactobacilli and its presence correlates with a reduced risk of preterm delivery, sexually transmitted infections and bacterial vaginosis [1, 2]. The ability of *L. crispatus* to colonize the vagina following oral or local administration has also been reported [3, 4]. Exopolysaccharide (EPS) production has been associated with beneficial effects of *L. crispatus* against vaginal pathogens and with its persistence in the gut environment [5, 6]. Furthermore, the production of bacteriocins, ribosomally synthesized peptides with antibiotic properties, may enhance bacterial fitness [7, 8]. Despite a large number of *L. crispatus* genomic sequences being available in public databases, to date only 23 are complete genomes. Furthermore, the *L. crispatus* mobilome, defined as the entire set of mobile genetic elements (MGEs), remains poorly annotated and characterized. Genomic variability among *L. crispatus* strains correlates with the site of isolation and is due to allelic variation in the EPS biosynthesis locus, in metabolic genes or to the presence of MGEs, including prophages and clustered regularly interspaced short palindromic repeats (CRISPRs) [9–11]. Lysogeny has been frequently observed in *L. crispatus* strains isolated from the vagina of healthy women [12], and some of these bacteriophages could be induced and had a lytic activity [13], but their nucleotide sequence has not been characterized. A more recent comparative genomic study identified prophage sequences in 90 out of 105 *L *. *crispatus* strains isolated from various sources, with complete prophage more likely in human strains [10]. In this work, we determined the complete genome sequence of *L. crispatus* strain M247, a newborn faecal isolate largely studied for its probiotic activity and characterized by a strong aggregation phenotype and adherence to intestinal mucus [14–19]. It has been reported that M247 has beneficial effects on intestinal inflammatory disorders [20–22], helps to counteract vaginal dysbiosis [23] and probably contributes to papilloma virus clearance [24]. The M247 genome sequence was obtained combining long Nanopore reads and Illumina reads and mobilome analysis revealed the presence of two novel MGEs: the integrative and mobilizable element (IME) Tn*7088* coding for a putative bacteriocin and the siphovirus prophage ΦM247.

## Methods

### Bacterial strains and growth conditions

*L. crispatus* strain M247 was isolated from faeces of human newborns [15]. Bacteria were grown in DeMan-Rogosa-Sharpe (MRS) broth (Oxoid) or in MRS supplemented with 1.5 % agar (BD Difco) in the presence of 5 % CO_2_ at 37 °C.

### Genomic DNA purification and quantification

Bacterial cells were grown at 37 °C in 250 ml of MRS broth until reaching middle exponential phase (OD_590_ of 1.9), and then harvested by centrifugation at 5 000 ***g*** for 30 min at 4 °C. High-molecular-weight genomic DNA was purified using a raffinose-based method [25, 26]. Briefly, a cell pellet was dry vortex-mixed for 2–3 min and incubated for 1 h at 37 °C in Protoplasting Buffer [20 % raffinose, 50 mM Tris/HCl (pH 8.0), 5 mM EDTA] containing 4 mg ml^−1^ lysozyme. Protoplasts were centrifuged at 5000 ***g*** for 5 min, resuspended in 15 ml of deionized H_2_O containing 100 µg ml^−1^ proteinase K (Merck) and 0.5 % SDS, and incubated for 30 min at 37 °C to obtain osmotic lysis. Then, 0.55 M NaCl was added and the mixture was incubated for 10 min at room temperature. High-molecular-weight DNA was purified three times with 1 volume of chloroform-isoamyl alcohol (24 : 1, v/v), precipitated in 0.6 volumes of ice-cold isopropanol and spooled on a glass rod. DNA was resuspended in 10-fold diluted saline-sodium citrate (SSC) 1× buffer, then adjusted to 1× SSC and maintained at 4 °C. The DNA solution was homogenized using a rotator mixer. DNA was quantified with a Qubit 2.0 Fluorometer (Invitrogen, Life Technologies) by using the Qubit dsDNA BR Assay Kit (Thermo Fisher Scientific) and results were confirmed via a spectrophotometer (Implen). DNA integrity and size were assayed by horizontal gel electrophoresis using 0.6 % Seakem LE (Lonza) agarose in 0.5× Tris borate EDTA running buffer.

### Illumina sequencing

Illumina sequencing was performed at MicrobesNG (University of Birmingham, UK) using a Nextera library preparation kit (Illumina) followed by sequencing on a NovaSeq 6000 device (Illumina) (2×250 bp paired-end sequencing). Illumina reads were trimmed using Trimmomatic v0.30 (https://github.com/usadellab/Trimmomatic) and analysed with FastQC v0.11.5 (https://www.bioinformatics.babraham.ac.uk/projects/fastqc/). Illumina read properties are reported in Table S1, available in the online version of this article.

### Nanopore sequencing

Nanopore sequencing was carried out essentially as already described [27, 28]. Briefly, sequencing reactions were carried out in 1.5 ml LoBind tubes (Sarstedt) using wide bore (∅1.2 mm) tips for DNA manipulation to reduce physical shearing. Genomic DNA was size selected with 0.5 volumes of AMPure XP beads (Beckman Coulter) according to the manufacturer’s instructions. Two micrograms of size-selected DNA was employed for library construction using the SQK-LSK 108 kit (Oxford Nanopore Technologies). Library preparation was performed following the manufacturer’s protocol with modifications: (i) incubation on rotator mix for 15 min; and (ii) the Library Loading Beads were not added. Finally, 1 µg of DNA library was loaded onto a R9.4 flow cell (FLO-MIN106) (Oxford Nanopore Technologies). A 21 h sequencing run was performed on a GridION device (Oxford Nanopore Technologies). Real-time base calling was performed with Guppy v3.2.6 (Oxford Nanopore Technologies), filtering out reads with a quality cut-off <Q7. Base called reads were analysed with NanoPlot v1.18.2 (https://github.com/wdecoster/NanoPlot). Nanopore read properties are reported in Table S1.

### Genome assembly and annotation

The M247 Nanopore reads were filtered to obtain 95× coverage taking 2.3 Mb as the genome size estimate using Filtlong v0.2.0 software (https://github.com/rrwick/Filtlong) with parameter *--target_bases* and assembled using Flye v2.7.1 (https://github.com/fenderglass/Flye). The resulting circular contig was polished with Medaka v0.7.1 (https://github.com/Nanoporetech/medaka) using all the Nanopore reads, followed by two polishing rounds with Pilon v1.22 (https://github.com/broadinstitute/pilon) using the Illumina reads. Assembly completeness was assessed with Bandage v0.8.1 (https://github.com/rrwick/Bandage), whereas assembly quality was evaluated with both Ideel (https://github.com/mw55309/ideel) and CheckM v1.1.3 (https://github.com/Ecogenomics/CheckM). Bwa v0.7.17 (https://github.com/lh3/bwa) and minimap2 v2.13 (https://github.com/lh3/minimap2) were used to align Illumina and Nanopore reads to the assembled genome, respectively. Aligned reads were visually inspected with Tablet v1.17.08.17 (https://github.com/cropgeeks/tablet) and used to further verify the assembled structure. The M247 genome was automatically annotated with the NCBI Prokaryotic Genome Annotation Pipeline (PGAP) v5.1 [29]. Default parameters were used for all software unless otherwise specified.

### Mobilome analysis

Mobilome analysis was performed as already described [30]: ICEfinder (https://bioinfo-mml.sjtu.edu.cn/ICEfinder/ICEfinder.html) was used to investigate the presence of bacterial integrative and conjugative elements (ICEs) and IMEs in the M247 genome, ISsaga (http://issaga.biotoul.fr/issaga_index.php) for insertion sequences (ISs), and PHASTER (http://phaster.ca), Virfam (http://biodev.cea.fr/virfam/) and Viridic (http://rhea.icbm.uni-oldenburg.de/VIRIDIC/) for prophages. The presence of CRISPRs was evaluated with CRISPRCasFinder (https://crisprcas.i2bc.paris-saclay.fr/CrisprCasFinder/Index). Analysis of antibiotic resistance genes analysis was performed using RGI (Resistance Gene Identifier) (v3.2.1) (https://card.mcmaster.ca/analyze/rgi), based on CARD (Comprehensive Antibiotic Resistance Database), with parameter ‘-loose_criteria=no’. DNA sequence analysis was performed with Artemis/ACT v17.0.1 (http://sanger-pathogens.github.io/Artemis/). Manual annotation of MGEs was carried out by blast homology searches of the databases available at the National Center for Biotechnology Information (NCBI) (https://blast.ncbi.nlm.nih.gov/Blast.cgi?PAGE=Proteins), and the Pfam protein family database (available under the InterPro consortium, https://www.ebi.ac.uk/interpro/search/sequence/). A transposon name was assigned by the Tn Registry website curators (https://transposon.lstmed.ac.uk/tn-registry). The *L. crispatus* complete genomes were downloaded from NCBI (https://www.ncbi.nlm.nih.gov/genome/browse/#!/prokaryotes/1815/) and the GenBank accession numbers are reported in Table S2.

### Mitomycin C induction and phage preparation

Mitomycin C induction and phage preparation were obtained essentially as previously reported [31]. Briefly, bacterial cells were grown in 600 ml of MRS broth until early exponential phase, and the culture was then split into three aliquots of which two were treated with 200 and 400 ng ml^−1^ of mitomycin C. After 21 h of incubation at 37 °C, EDTA was added at a final concentration of 10 mM and the samples were centrifuged twice at 5 000 ***g*** for 40 min at 4 °C in 50 ml tubes to eliminate bacterial cells and cellular debris. The recovered supernatants were transferred into six-polyallomer centrifuge tubes and ultracentrifuged at 20 000 ***g*** for 2 h at 10 °C in an Optima L-90K ultracentrifuge with the SW 32 Ti rotor (Beckman Coulter). The phage pellets were resuspended in 350 µl of TM buffer (50 mM Tris/HCl, 10 mM MgSO_4_) and the phage preparations were stored at 4 °C.

### PCR and sequencing

PCR and direct PCR sequencing were carried out as previously described [32–35]. Oligonucleotide primers and their properties are reported in Table S3. The circular forms of Tn*7088* and ΦM247 were detected using divergent primers directed at the ends of the elements, while reconstitution of the target sites using primers directed at the chromosomal junction fragments as reported [36, 37]. The following templates were used: (i) the purified high-molecular-weight DNA and (ii) the phage preparations from mitomycin C-treated and untreated M247 cultures. Briefly, quantitative PCR (qPCR) was carried out with the KAPA SYBR FAST qPCR kit Master Mix Universal (2×) (Merck) on a LightCycler 1.5 apparatus (Roche Diagnostics). The real-time PCR mixture contained, in a final volume of 20 µl, 1× KAPA SYBR FAST qPCR mix, 5 pmol of each primer, and 20 ng of bacterial genomic DNA or 2 µl of phage preparation as starting template. The thermal profile was an initial 3 min denaturation step at 95 °C followed by 40 cycles of repeated denaturation (0 s at 95 °C), annealing (20 s at 62 °C) and polymerization (30 s at 72 °C). The temperature transition rate was 20 °C s^–1^ in the denaturation and annealing steps and 5 °C s^–1^ in the polymerization step. Primer pair IF1487/IF1488 amplifying a 350 bp fragment, and primer pair IF1513/IF1514 amplifying a 426 bp fragment were used for quantification of circular forms of Tn*7088* and ΦM247, respectively; while IF1349/IF1350 amplifying a 227 bp fragment and IF1511/IF1512 amplifying a 133 bp fragment were used for free locus quantification of Tn*7088* and ΦM247, respectively; a 292 bp fragment of the chromosomal *gyrB* gene, obtained with primers IF1352/IF1353, was used to standardize results. A standard curve for the *gyrB* gene of *L. crispatus* M247 was built by plotting the threshold cycle against the number of chromosome copies using serial dilutions of chromosomal DNA with known concentration. Melting curve analysis was performed to differentiate the amplified products from primer dimers. A t-test was used to assess the statistical significance of differences in quantification values of excised forms.

## Results

### The M247 genome

**Fig. 1.**
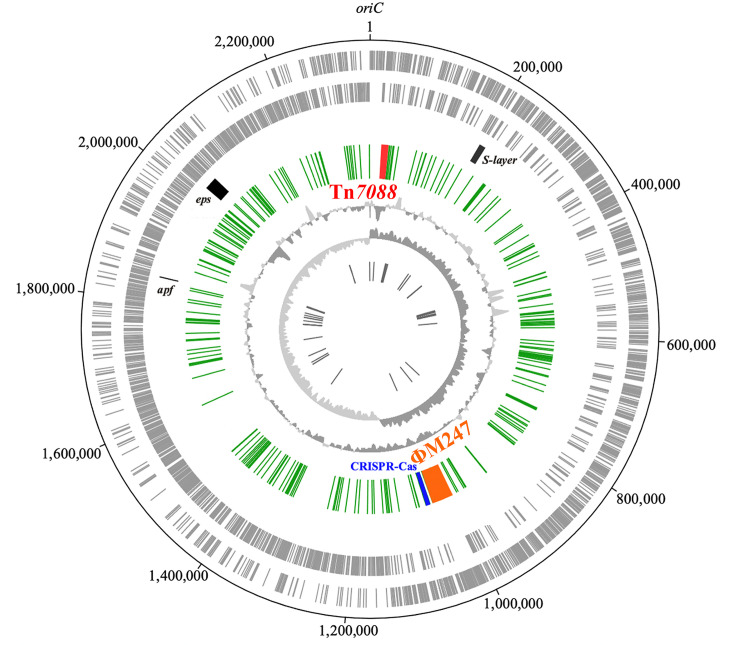
Circular representation of the *L. crispatus* M247 genome. Circle 1 (outer) and circle 2 show the predicted coding regions located on the plus and minus strands, respectively. S-layer, aggregation promoting factor (*apf*) and EPS biosynthesis (*eps*) loci are reported as black blocks. The third circle represents the mobilome including the 14 105 bp Tn*7088* IME (red block), the 42 510 bp ΦM247 prophage (orange block), the CRISPR-Cas locus (blue block) and the 226 ISs (green ticks). The fourth and the fifth circles show GC content and GC skew, respectively. The innermost circle indicates RNA genes. The image was created using Artemis DNA-Plotter (v17.0.1).

**Fig. 2.**
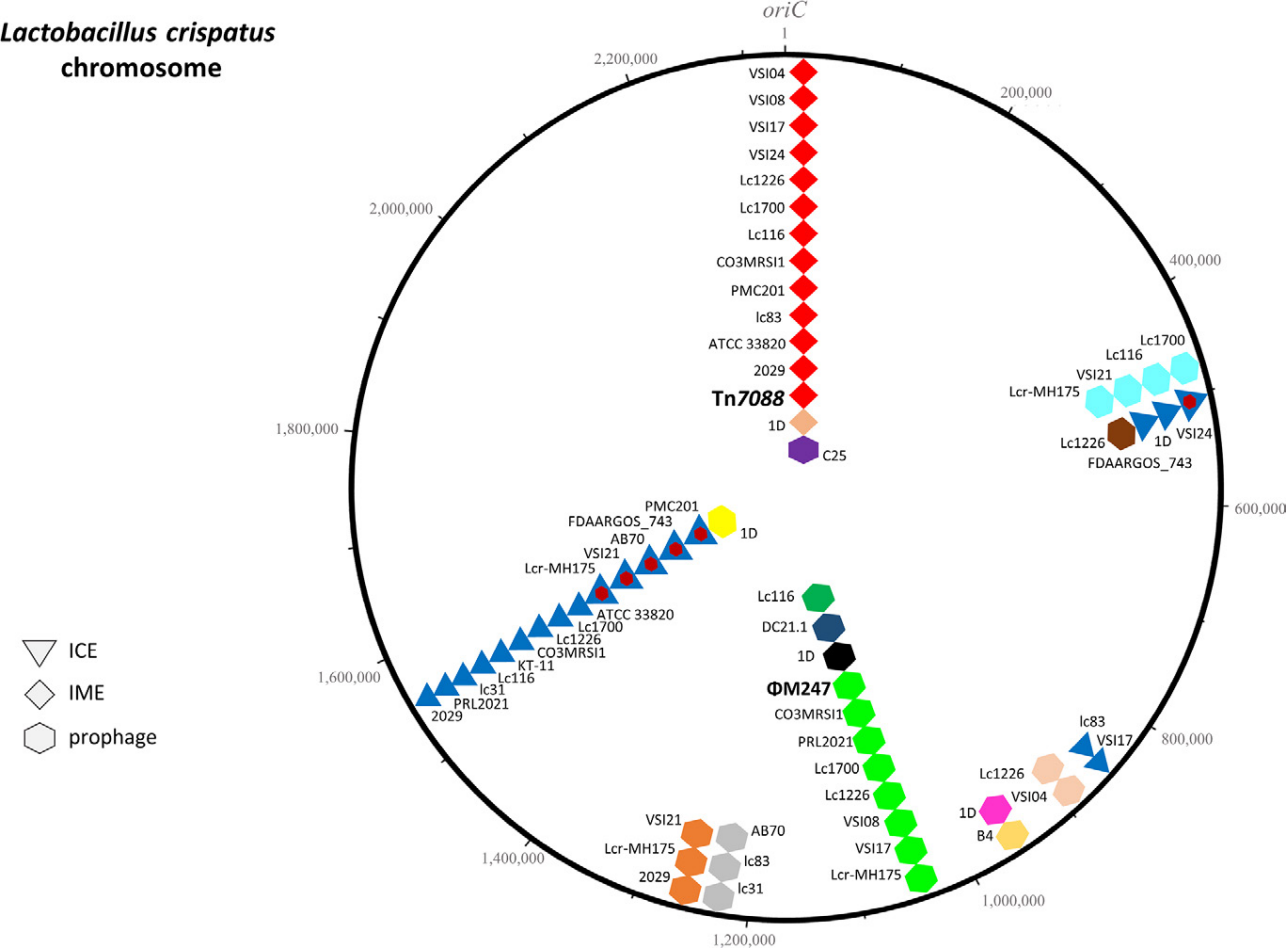
Integration sites of MGEs in the *L. crispatus* chromosome. Analysis of the 23 *L *. *crispatus* complete genomes available in the NCBI database (last accessed July 2023) revealed that MGEs integrate at 10 chromosomal sites. The *L. crispatus* mobilome consists of one ICE, two IMEs, 13 different prophages, and a composite element constituted by a prophage inserted in an ICE. Bacterial chromosome is represented by a circle, and the origin of replication (*oriC*) is indicated; nucleotides reported on the map refer to the M247 strain used as a reference. Triangles, diamonds and hexagons on the circle indicate the insertion sites of ICEs, IMEs and prophages, respectively. Homologous elements are depicted with symbols of the same colour. IME Tn*7088* and prophage ΦM247 found in strain M247 are indicated with their names, while non-annotated elements are indicated with the names of the strain harbouring them. GenBank accession numbers of the genome sequences are reported in Table S2.

**Fig. 3.**
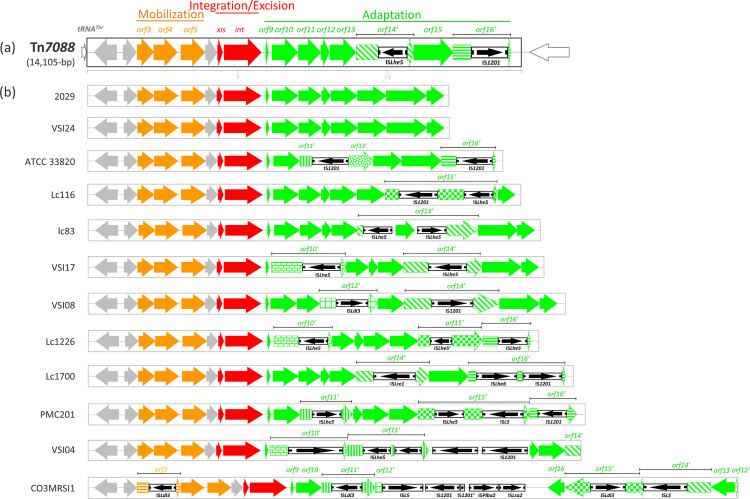
(**a**) Structure of *L. crispatus* IME Tn*7088* and (**b**) schematic comparison of Tn*7088* with other *L. crispatus* Tn*7088*-like elements. (**a**) Tn*7088* is a 14 105 bp long IME, and contains 16 ORFs and two ISs. ORFs and their direction of transcription are represented by arrows, and annotated ORFs are indicated. The element shows a modular organization characterized by three modules: mobilization, integration/excision and adaptation (indicated by solid bars). ISs are reported as thinner, black, boxed arrows. IS*Lhe5* disrupts *orf14*, while IS*1201* disrupts *orf16*. Chromosomal genes flanking Tn*7088* are represented by white arrows. The scale is in kilobases. (**b**) IME Tn*7088* is compared with 12 *L *. *crispatus* Tn*7088*-like elements present in complete genomes available in public databases. Disrupted ORFs are reported as pattern filled arrows. Non-annotated elements are indicated with the names of the strains harbouring them. Size varies from 11 678 bp for the 2029 element to 22 175 bp for the CO3MRSI1 element. Genome sequence GenBank accession numbers are reported in Table S2.

**Fig. 4.**
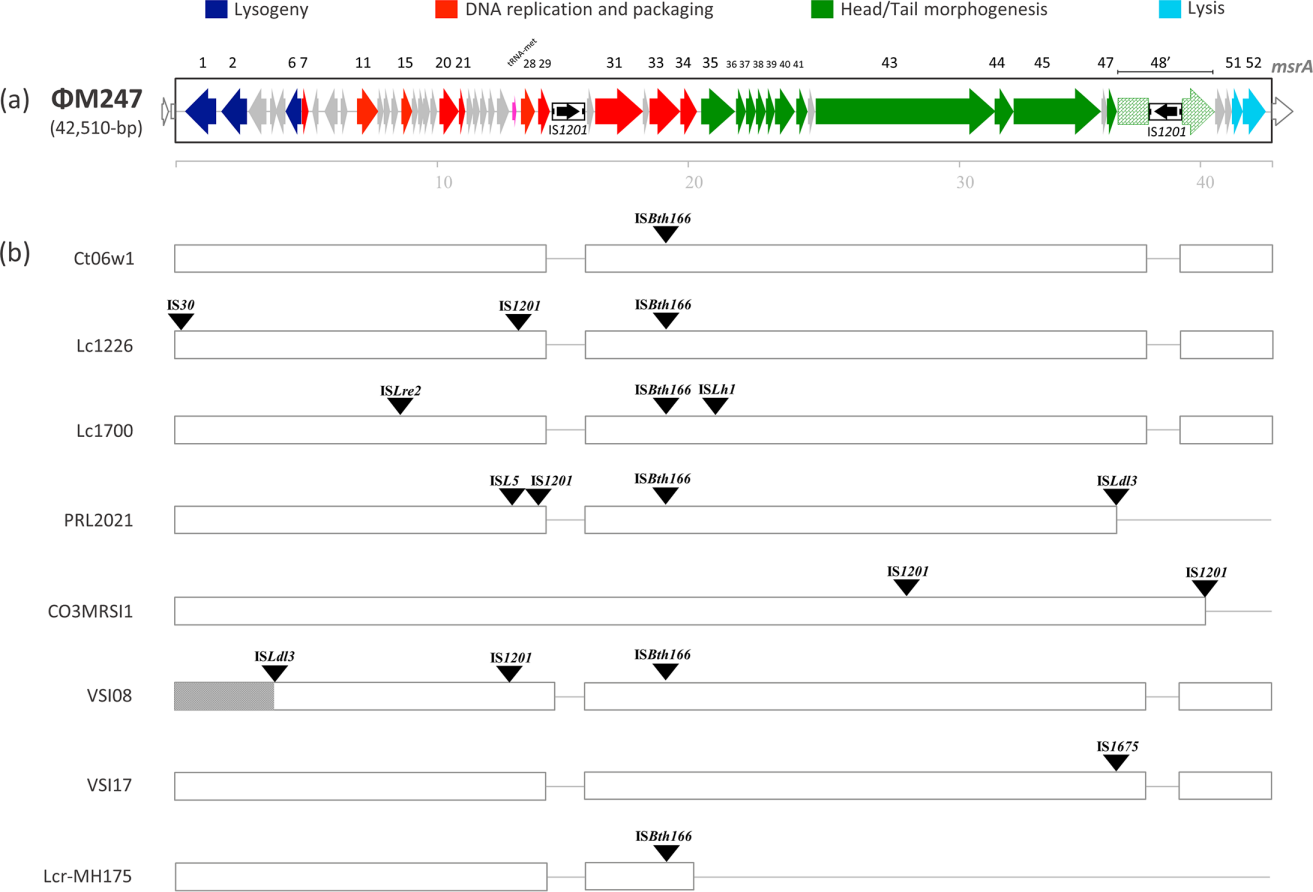
(**a**) Structure of *L. crispatus* prophage ΦM247 and (**b**) schematic comparison of ΦM247 with other ΦM247-like prophages. (**a**) ΦM247 is a 42 510 bp long prophage, and contains 52 ORFs and two copies of the IS*1201* one of which disrupts *orf48* (pattern filled arrow). ORFs and their direction of transcription are represented by arrows, while annotated ORFs are indicated only by their numbers. ISs are reported as thinner, black, boxed arrows. Chromosomal genes flanking the ΦM247 insertion site are represented by white arrows. The scale is in kilobases. (**b**) The 42 510 bp ΦM247 prophage is compared with the siphovirus prophage Ct06w1 (GenBank BK036340) and seven *L *. *crispatus* ΦM247-like prophages present in complete genomes. For a better alignment of the sequences, the elements were devoid of additional integrated IS elements indicated by solid triangles, while the deletions are represented by lines. Size varies from 37 638 bp for the CO3MRSI1 element to 44 067 bp for the Lc1700 element. Non-annotated elements are indicated with the names of the strains harbouring them. The element of strain VSI08 contains a 3 391 bp inversion at the 5′ end depicted as a grey box. Genome sequence GenBank accession numbers are reported in Table S2.

Sequence analysis showed that the M247 genome is organized in one circular chromosome 2 336 109 bp in length, with an average GC content of 37.04 % (Fig. 1). The genome contains 2303 ORFs which are equally distributed on both strands (1161 on sense and 1142 on antisense). An annotation with prediction of a biological function was possible for 1962 ORFs. rRNA genes are grouped into four rRNA operons, 28 out of the 65 tRNA genes are not adjacent to rRNA operons, and three structural RNAs are also present: (i) tRNA-like/mRNA-like RNA, (ii) signal recognition particle RNA and (iii) ribonuclease P RNA. The M247 genome contains a single copy of the *apf* gene [18], spanning nucleotides 1 837 153–1 837 824, while the already described *S-layer* locus (GenBank no. AY941197) spans nucleotides 197 946–209 531, and includes the paralogous genes *slpA* and *slpB* transcribed in opposite directions and spaced by a 4 624 bp fragment. A 79 695 bp EPS biosynthesis gene cluster, at nucleotides 1 956 291–2 035 986, contains 57 ORFs, 52 of which have the same direction of transcription and are potentially involved in EPS biosynthesis. Five conserved genes predicted to encode a transcriptional regulator, a polymerization and chain length determination protein, a tyrosine-protein kinase, a protein-tyrosine phosphatase, and the priming glycosyltransferase are present at the 5′ end of the locus as reported in other *L. crispatus* strains [9]. Downstream of this conserved region, nine genes encoding (i) five glycosyltransferases, (ii) a UDP-*N*-acetylglucosamine, (iii) an acyltransferase, (iv) a polysaccharide polymerase and (v) a flippase are probably involved in the synthesis of EPS repeating units, polymerization and export. The remaining genes, as in other lactobacilli, probably contribute to EPS biosynthesis, for example through the generation of activated sugar precursors and the chemical decoration of the EPS [38]. A search carried out on the 23 publicly available *L. crispatus* complete genomes showed that the *L. crispatus* mobilome includes, in addition to plasmids, ISs and CRISPRs, prophages, ICEs and IMEs which are currently not annotated (Fig. 2). Analysis indicates the presence of 10 chromosomal sites for the integration of these elements. The M247 mobilome accounts for 14 % (328 388 bp) of the whole genome and includes two novel genetic elements, IME Tn*7088* (Fig. 3), and the siphovirus prophage ΦM247 (Fig. 4), three CRISPRs and 226 ISs belonging to 14 different families (Table S4). No plasmids were detected. Three CRISPR loci were found in the M247 mobilome: (i) locus 1 contains five 23 bp direct repeats (DRs) interspersed by four spacers; (ii) locus 2 contains six 36 bp DRs interspersed by five spacers, and is paired to the CRISPR-associated (Cas) *cas9*, *cas1*, *cas2* and *csn2* genes constituting a type II-A CRISPR-Cas system [39] (Fig. 1); and (iii) locus 3 contains two 25 bp DRs interspersed by one spacer. Despite the M247 genome being rich in ISs, which make up 11.16 % (260 880 bp) of its length, only 10 ORFs are disrupted by ISs, of which two are located on Tn*7088* and one on ΦM247. The transposase gene of 26 ISs is truncated or presents a frameshift mutation (Table S4).

### The integrative and mobilizable element Tn*7088*

Tn*7088* is 14 105 bp long, spans nucleotides 21 914–36 018 of the M247 genome and has an average GC content of 30.97 %. Tn*7088* contains 16 ORFs, of which 15 have the same direction of transcription, two have a GTG alternative start codon and two are disrupted by different ISs (Fig. 3). Manual homology-based annotation attributed a putative function to 14 out of 16 ORFs (Table 1). Tn*7088* has a typical IME modular organization [40] composed of (i) a mobilization module where *orf3* and *orf4* code for FtsK homologous proteins and *orf5* for a relaxase; (ii) an integration/excision module constituted by *orf7* and *orf8*, coding for a putative excisionase and an integrase, respectively; and (iii) an adaptation module (*orf9* to *orf16*) homologous to the listeriolysin S (*lls*) locus of *Listeria monocytogenes* [41, 42] (Fig. 3a, Table 1). The 44 aa Orf9 is homologous to LlsA, the pro-peptide listeriolysin S family of thiazole/oxazole-modified microcins (TOMM) class I bacteriocin, and contains a 13 aa long serine/threonine/cysteine-rich motif. Orf10–Orf11 are homologous to LlsG–LlsH that constitute an ATP-binding cassette transporter, whereas Orf12 is homologous to the hypothetical protein LlsX. Orf13 is homologous to the LlsB dehydrogenase, while Orf14 and Orf15 are homologous to the LlsY and LlsD cyclodehydratases, respectively. Finally, Orf16 is homologous to the metalloprotease LlsP. The NCBI database of 95 347 complete microbial genomes (accessed in July 2023) was interrogated using Tn*7088* DNA sequence as a query. In 12 out of 23 *L *. *crispatus* complete genomes, Tn*7088*-like elements, ranging in length from 11 678 bp for strain 2029 to 22 175 bp for strain CO3MRSI1, were present. Sequence comparison showed that the mobilization and integration/excision modules are essentially conserved, while variability in the adaptation module is introduced by the integration of distinct ISs disrupting different ORFs (Fig. 3b). It is of note that only the Tn*7088*-like elements carried by strains 2029 and VSI24 contain an intact adaptation module. In the CO3MRSI1 element a copy of IS*Ldl3* disrupts *orf3* in the mobilization module, whereas the adaptation module carries a DNA inversion spanning the 3′ end of *orf12* to *orf16*.

**Table 1.** Annotated ORFs of IME Tn*7088*

ORF (aa)*	Annotation and comments (reference)	Homologous protein			Pfam domain (aa)‡ [E value]
		Protein ID/origin [E value]†	No. of amino acids identical/total	No. of amino acids similar/total	
*orf1* (254)	Transcriptional regulator, putative [63]				HTH_3 (6–66) [1.0e −12]
*orf3* (183)	Cell division protein FtsK, putative [64]				
*orf4* (264)	Cell division protein FtsK [64]				FtsK_SpoIIIE (2–109) [1.1e-05]
*orf5* (273)	Relaxase [65]				Rep_trans (133–273) [1.1e-19]
*orf7* (59)	Excisionase, putative				
*orf8* (409)	Tyrosine-type DNA integrase [66]				Phage_integrase (183–397) [9.8e-14]
*orf9* (44)	Listeriolysin S family TOMM class I bacteriocin [54]	WP_071661762.1/* L. monocytogenes* [3e-04]	12/26 (46 %)	14/26 (53 %)	
*orf10* (284)	ABC transporter: ATP-binding protein [54], similar to *L. monocytogenes* LlsG	WP_003724649.1/* L. monocytogenes* [5e-69]	111/284 (39 %)	182/284 (64 %)	ABC_tran (21–150) [7.6e-22]
*orf11* (251)	ABC transporter: permease [54], similar to *L. monocytogenes* LlsH	WP_003730942.1/* L. monocytogenes* [2e-70]	96/238 (40 %)	160/238 (67 %)	ABC2_membrane (5–212) [3.2e-12]
*orf13* (292)	SagB dehydrogenase family [54], similar to *L. monocytogenes* LlsB	WP_003730944.1/* L. monocytogenes* [6e-95]	135/291 (46 %)	192/291 (65 %)	Nitroreductase (105–285) [5.8e-13]
*orf14*’ (314)	Listeriolysin S biosynthesis cyclodehydratase [42], similar to *L. monocytogenes* LlsY, disrupted by IS*Lhe5*	WP_010958846.1/* L. monocytogenes* [3e-72]	128/306 (42 %)	192/306 (62 %)	
*tnp* (285)	IS*Lhe5* transposase [67]	WP_012211839/ * L. helveticus* [0.0]	240/285 (84 %)	259/285 (90 %)	
*orf15* (438)	YcaO-like cyclodehydratase family [54], similar to *L. monocytogenes* LlsD	WP_003740559.1/* L. monocytogenes* [3e-172]	240/440 (55 %)	312/440 (70 %)	YcaO (70–407) [1.5e-28]
*orf16’* (195)	CPBP intramembrane metalloprotease family [42], similar to *L. monocytogenes *, LlsP disrupted by IS*1201*	WP_226989712.1/* L. monocytogenes* [7e-31]	56/147 (38 %)	92/147 (62 %)	CPBP (5–170) [0.045]
*tnp* (408)	IS*1201* transposase [68]	P35880/* L. helveticus* [0.0]	333/368 (90 %)	352/368 (95 %)	

*The number of amino acids of the predicted protein is shown in parentheses, and disrupted ORFs are indicated with an apostrophe.

†Determined by compositional matrix adjustment.

‡Numbers in parentheses indicate the part of the predicted protein with homology to the Pfam domain.

### Prophage ΦM247

The 42 510 bp ΦM247 prophage spans nucleotides 1 001 143–1 043 652 of the M247 genome, carries 52 ORFs, has a GC content of 35.18 % and was classified as a siphovirus phage. Manual homology-based annotation with functional prediction of the hypothetical gene product was possible only for 27 out of 52 ORFs, including genes for phage structural proteins (*orf35* to *orf48*), DNA replication (*orf7, orf11*, *orf15*, *orf20*, *orf21*, *orf28*) and packaging (*orf29* to *orf34*), lysogeny (*orf1*, *orf2*, *orf6*) and lytic cycle-related proteins (*orf51*, *orf52*) (Table 2, Fig. 4a). Furthermore, ΦM247 contains a methionyl-tRNA gene which might contribute to modulate the tRNA pools of the bacterium, improving the translation efficiency of viral genes [43, 44]. Two copies of IS*1201* are also present, one of which disrupts *orf48,* predicted to encode the tail protein. Homology searches of the NCBI databases using nucleotide blast (accessed in July 2023) showed that ΦM247 is highly homologous (99.98 % identity) to the ct06w1 siphovirus prophage characterized during the metagenomic sequencing of a human vaginal fornix sample [45]. In addition, ΦM247-like prophages are present only in seven *L *. *crispatus* complete genomes, namely Lc1226, PRL2021, Lc1700, CO3MRSI1, VSI08, VSI17 and Lcr-MH175, out of the 95 347 complete microbial genomes of the NCBI database (accessed in July 2023). Variability in ΦM247-like prophage sequences is due to the integration of distinct ISs in different positions except for strains CO3MRSI1, PRL2021 and Lcr-MH175 where a 3′ end deletion of 3.4, 4.6 and 22.14 kb, respectively, was also present (Fig. 4b).

**Table 2.** Annotated ORFs of ΦM247

ORF (aa)*	Annotation and comments (reference)	Virfam homologous protein (identity) [E value or HHsearch probability]†	Homologous protein ID/origin No. of amino acids identical/total [E value]‡	Pfam domain (aa)§ [E value]
*orf1* (407)	Tyrosine-type DNA integrase [66]		DAW29718.1/*Siphoviridae *sp. isolate ct06w1 407/407 (100 %) [0.0]	Phage_integrase (180–373) [1.8e-25] Phage_int_SAM_5 (32–164) [1.8e-05]
*orf2* (334)	Abortive infection protein [69]		DAW29696.1/*Siphoviridae *sp. isolate ct06w1 334/334 (100 %) [0.0]	Abi_2 (31–239) [1.5e-36]
*orf6* (208)	Repressor protein CI [70]		DAW29717.1/*Siphoviridae *sp. isolate ct06w 208/208 (100 %) [9e-27]	Peptidase_S24 (86–202) [3.1e-26]
*orf7* (71)	Helix-turn-helix XRE-family-like protein [63]		DAW29695.1/*Siphoviridae *sp. isolate ct06w 74/74 (100 %) [9e-56]	
*orf11* (285)	DNA polymerase B		DAW29710.1/*Siphoviridae *sp. isolate ct06w1 285/285 (100 %) [1e-158]	HTH_36 (24–74) [4.4e-05]
*orf15* (145)	HNH endonuclease [71]		DAW29694.1/*Siphoviridae *sp. isolate ct06w1 145/145 (100 %) [3e-111]	HNH_3 (66–112) [7.5e-10]
*orf20* (248)	Phage antirepressor KilAC domain-containing protein [72]		DAW29715.1/*Siphoviridae *sp. isolate ct06w1 247/248 (99 %) [0.0]	AntA (17–85) [5.7e-19] ANT (124–236) [8.9e-32]
*orf21* (74)	Restriction alleviation protein, putative		DAW29742.1/*Siphoviridae *sp. isolate ct06w1 73/73 (100 %) [2e-55]	
*orf27*	tRNA-Met			
*orf28* (176)	HNH endonuclease [73]		DAW29714.1/*Siphoviridae *sp. isolate ct06w1 176/176 (100 %) [2e-138]	HNH (88–131) [8.1e-08]
*orf29* (156)	Phage terminase, small subunit [74]		WP_060463559.1/*L.crispatus* 154/156 (99 %) [1e-55]	Terminase_4 (29–140) [4.3e-16]
*tnp* (392)	IS*1201* transposase [68]		P35880/* L. helveticus * 333/368 (90 %) [0.0]	
*orf31* (624)	Phage terminase, large subunit [74]	Phi adh phage TermL (60%) [0]	DAW29733.1/*Siphoviridae *sp. isolate ct06w1 624/624 (100 %) [0.0]	Terminase_1 (100–587) [1.5e-50]
*orf33* (392)	Phage portal protein [75]	Phi adh phage Portal (54%) [100%]	WP_060464314.1/*L.crispatus* 392/392 (100 %) [3e-66]	Phage_portal (47–354) [4.9e-39]
*orf34* (228)	ATP-dependent Clp protease [76]		DAW29711.1/*Siphoviridae *sp. isolate ct06w1 228/228 (100 %) [7e-174]	CLP_protease (32–175) [2.0e-33]
*orf35* (452)	Phage major capsid protein	DT1 phage MCP (36%) [100%]	DAW29706.1/*Siphoviridae *sp. isolate ct06w1 452/452 (100 %) [0.0]	Phage_capsid (132–421) [2.8e-17]
*orf36* (129)	Phage head-tail adaptor	Phi adh phage Ad1 (42%) [100%]	DAW29705.1/*Siphoviridae *sp. isolate ct06w1 129/129 (100 %) [2e-98]	
*orf37* (121)	Phage head closure knob	Phi adh phage Hc1 (34%) [100%]	DAW29704.1/*Siphoviridae *sp. isolate ct06w1 121/121 (100 %) [8e-94]	Phage_H_join (12–110) [0.00065]
*orf38* (132)	Phage type I neck protein	DT1 phage Ne1 (36%) [100%]	DAW29703.1/*Siphoviridae *sp. isolate ct06w1 132/132 (100 %) [4e-101]	HK97-gp10_like (6–98) [0.00044]
*orf39* (124)	Phage tail completion protein	Phi adh phage Tc1 (24%) [100%]	DAW29702.1/*Siphoviridae *sp. isolate ct06w1 124/124 (100 %) [4e-94]	
*orf40* (258)	Phage major tail protein [77]	DT1 phage MTP (30%) [3e-20]	DAW29701.1/*Siphoviridae *sp. isolate ct06w1 258/258 (100 %) [0.0]	Phage_TTP_1 (8–211) [1.7e-42]
*orf41* (137)	Phage tail assembly chaperone [77]		DAW29701.1/*Siphoviridae *sp. isolate ct06w1 137/137 (100 %) [4e-103]	Phage_TAC_3 (7–122) [1.5e-11]
*orf43* (2339)	Phage tail tape measure protein [77]		DAW29700.1/*Siphoviridae *sp. isolate ct06w1 2338/2339 (99 %) [0.0]	PhageMin_Tail (315-532) [1.1e-38]
*orf44* (253)	Phage distal tail protein [77]		DAW29699.1/*Siphoviridae *sp. isolate ct06w1 252/253 (99 %) [0.0]	
*orf45* (1135)	Phage tail protein [77]		DAW29698.1/*Siphoviridae *sp. isolate ct06w1 1135/1135 (100 %) [0.0]	Prophage_tail (73–441) (744–824) [7.2e-13]
*orf47* (127)	BppU family phage baseplate upper protein		WP_005719141.1/* Lactobacillus crispatus * 127/127 (100 %)	BppU_N (1–120) [5.6e-09]
*orf48’* (795)	Phage tail protein [78], disrupted by IS*1201*		DAW29725.1/*Siphoviridae *sp. isolate ct06w1 795/795 (100 %) [0.0]	Bppu_N (3–164) [3.7e-08] Lipase_GDSL (575–776) [8.4e-12]
*tnp* (392)	IS*1201* transposase [68]		P35880/* L. helveticus * 333/368 (90 %) [0.0]	
*orf51* (142)	Phage holin LLH family [79]		DAW29722.1/*Siphoviridae *sp. isolate ct06w1 142/142 (100 %) [8e-107]	Phage_holin_6_1 (3–110) [1.3e-05]
*orf52* (294)	Cpl1 lysin [80]		DAW29716.1/*Siphoviridae *sp. isolate ct06w1 294/294 (100 %) [0.0]	Glyco_hydro_25 (9–196) [1.8e-21]

*The number of amino acids of the predicted protein is shown in parentheses, and disrupted ORFs are indicated with an apostrophe.

†Virfam performs PSI-blast on the ACLAME database, and the HHsearch probability is provided when PSI-blast search does not detect homology.

‡Determined by compositional matrix adjustment.

§Numbers in parentheses indicate the part of the predicted protein with homology to the Pfam domain.

### Quantification of Tn*7088* and ΦM247 excised forms and reconstituted *attB* sites

PCR and sequencing analysis carried out on M247 genomic DNA showed that the Tn*7088* IME is able to excise from the bacterial chromosome producing a circular form where the left and right ends are joined by a 90 bp sequence (*att*Tn). Excision of the element reconstitutes the chromosomal 79 bp attachment site (*att*B). Upon integration into the M247 chromosome, Tn*7088* is flanked by *att*L and *att*R, identical to *att*Tn and *att*B, respectively. *att*L-*att*Tn contain 11 nt insertions and 12 nt changes compared to *att*R-*att*B (Fig. S1). *att*R-*att*B contain the last 11 nt of the threonine-tRNA gene (LQF73_00105). In liquid culture of M247, the circular form of Tn*7088* was present at a concentration of 4.32×10^−5^±2.17×10^−7^ copies per chromosome, whereas the reconstituted *att*B site was at 6.65×10^−4^±1.32×10^−6^ copies per chromosome. Also, prophage ΦM247 produces an excised form, where the left and right ends are joined by *att*P, restoring the *att*B insertion site. *att*P is 138 bp long and is identical to *att*R, while *att*B is 139 bp long and is identical to *att*L. *att*R-*att*P differ from *att*L-*att*B by 15 nt changes and 1 nt nucleotide deletion. *att*L-*att*B of ΦM247 includes 96 nt at the 5′ end of the peptide-methionine (S)-S-oxide reductase encoding gene *msrA* (LQF73_05265). The excised form of the phage genome was present at a concentration of 3.90×10^−5^±9.43×10^−6^ copies per chromosome, whereas the reconstituted *att*B site was at 2.48×10^−5^±7.18×10^−7^ copies per chromosome.

### ΦM247 excised forms are not enriched upon mitomycin C exposure

Liquid cultures of M247 were treated with mitomycin C at a final concentration of 200 and 400 ng ml^−1^, and culture supernatants were recovered and concentrated by sequential centrifugation and ultracentrifugation steps without filtering to minimize particle breakage. ΦM247 excised forms were detected and quantified with qPCR on concentrated supernatants. The number of excised forms per millilitre was essentially the same (*P*=0.09) in phage preparations obtained from the mitomycin C-treated cultures (mean value 2.14×10^2^±1.07×10^2^) and the untreated control culture (7.66×10^2^±6.17×10^2^), suggesting that, under our experimental conditions, mitomycin C does not induce excision of ΦM247.

## Discussion

In this study, complete genome sequence analysis allowed us to define the M247 mobilome which includes: (i) the novel IME Tn*7088*, (ii) the novel ΦM247 prophage, (iii) three CRISPRs and (iv) 226 ISs. More than 10 % of the M247 genome length is constituted by ISs, which only interrupt 10 predicted coding sequences, suggesting that there is a selective pressure against gene disruption. Interestingly, three out of 10 interrupted genes belong to ΦM247 and Tn*7088*, which are probably less subject to selective constraints. The presence of many ISs in the M247 genome possibly confers genome plasticity, favouring chromosomal rearrangements and duplications mediated by sequence homology [46]. ΦM247 was capable of excision from the bacterial chromosome, but could not be induced by mitomycin C. Some active prophages are not responsive to genotoxic drugs such as mitomycin C, but are induced by other triggering factors [47–49]. Further investigations will clarify if also ΦM247 is responsive to triggering factors other than mitomycin C. Genomic analysis showed that prophages homologous to ΦM247 are present in seven other complete genomes belonging only to *L. crispatus *, suggesting host specificity of ΦM247 [50]. Tn*7088* contains three functional modules: (i) mobilization, (ii) integration/excision and (iii) adaptation. In the mobilization module *orf3*, *orf4* and *orf5* encode FtsK homologous proteins and a relaxase, respectively, which probably constitute the relaxosome [51]. The integration/excision module contains *orf7* and *orf8* coding for a putative excisionase and for a site-specific integrase that belongs to the family of tyrosine recombinases, as reported for many IMEs [52]. IMEs integrate into the bacterial genome and are capable of intracellular transposition to a new genomic site or intercellular transposition to a new host using *in trans* the conjugation machinery of a helper ICE or conjugative plasmid [40, 53]. The presence of circular intermediates of Tn*7088* was detected by PCR analysis, proving that the integration/excision module is functional and the element has the potential to be horizontally transferred. Biosynthetic gene clusters for the synthesis of class I bacteriocins are widely distributed in both Gram-positive and Gram-negative bacteria [54]. These bacteriocin gene clusters were reported in *L. crispatus *, although the genomic location was not indicated [55, 56]. Recently, it has been demonstrated that listeriolysin S does not contribute to *L. monocytogenes* virulence and tissue damage, but instead targets exclusively prokaryotic cells, playing a role in modulation of the host microbiota [57–59] and acting via a cell-to-cell contact mechanism of action [60]. We found that a bacteriocin gene cluster homologous to that of the *L. monocytogenes* listeriolysin S constitutes the adaptation module of the IME Tn*7088* and Tn*7088*-like elements, and possibly mediates an interaction with other bacteria of the microbiota. To the best of our knowledge, Tn*7088* is the first example of a mobile element containing a predicted biosynthetic gene cluster for a class I bacteriocin in *L. crispatus *. The Tn*7088* adaptation module contains an *orf* coding for a bacteriocin pro-peptide carrying a serine/threonine/cysteine-rich motif which probably acts as target site in post-translational modifications [61] and *orf*s coding for all enzymes required for post-translational modifications of the bacteriocin pro-peptide [62]. However, the disruption of various ORFs of Tn*7088* and Tn*7088*-like elements by integration of ISs leaves an open question regarding whether the adaptation module does or does not encode for a functional bacteriocin. Further investigations are needed to elucidate the contribution of Tn*7088* to the niche-adaptive traits of the M247 host.

## Supplementary Data

Supplementary material 1
