# Supplementary material for: The mobilome of Lactobacillus crispatus M247 includes two novel genetic elements: Tn7088 coding for a putative bacteriocin and the siphovirus prophage ΦM247
Source: Microb Genom. 2023 Dec 12;9(12):001150. doi: 10.1099/mgen.0.001150 (PMC10763512; doi:10.1099/mgen.0.001150)

Table S1. General statistics M247 Nanopore and Illumina reads.

	Nanopore reads		Illumina reads	
	**Raw**	**Filtered**	**R1**	**R2**
Mean read length	13,483.4	41,433.7	209.4	194.4
Mean read quality	11.8	13.9	33.8	30.2
Median read length	7,705.0	36,622.0	250.0	220.0
Median read quality	12.0	13.9	35.0	29.8
Number of reads	153,079	5,275	268,663	268,663
Read length N50	25,587	44,883	251	225
Total bases (bp)	2,064,031,022	218,562,625	56,246,560	52,216,531

Table S2. GenBank accession numbers of the *L. crispatus* complete genomes used for the analysis.

Strain name	GenBank accession no.
AB70	CP026503
CO3MRSI1	CP033426
FDAARGOS_743	CP046311
PRL2021	CP058996
1D	CP047415
C25	CP047142
B4	CP059140
DC21.1	CP039266
ATCC 33820	CP072197
Lc1226	CP083392
Lc1700	CP083389
Lc116	CP083393
2029	CP079206
PMC201	CP076522
KT-11	AP025162
lc83	CP061005
lc31	CP061006
Lcr-MH175	CP114552
VSI04	CP118947
VSI08	CP118087
VSI17	CP118077
VSI21	CP118069
VSI24	CP118067

Table S3. Oligonucleotide primers.

Name	Sequence (5' to 3')	Position on M247 genome
IF1349	CGGGTAAGACAACGAAGAGT	21,831 – 21,850
IF1350	TCCAACGCCTGTTAAATCACTA	36,239 – 36,218
IF1352	GAGTTGGGGCTTCAGTAGTT	4,645 – 4,664
IF1353	AGTGTCTTTACGCTTGTCAGTA	4,937 – 4,916
IF1487	TGTGCCACACGGTTTCTAGA	35,945 – 35,964
IF1488	TTTATAGTACCTTTGCCACACAA	22,110 – 22,088
IF1511	GAAATAAAATGGGATACATCAGGT	1,000,932 – 1,000,955
IF1512	CCACCAGTATAACCAGAAACTA	1,043,711 – 1,043,690
IF1513	CCGTAAGGAGGAGATGCTAA	1,001,173 – 1,001,154
IF1514	CGCTCTAGGGGTAAAACTCTA	1,043,397 – 1,043,417

Table S4. Insertion Sequences in the *L. crispatus* M247 genome.

**IS family**	**IS name**	**Copies***
IS*256*	IS*1201*	83 (9)
IS*982*	IS*Lhe5*	9 (2)
	IS*Lh1*	15
IS*3* sub-group IS*150*	IS*Lhe6*	7 (2)
	IS*Enfa5*	10 (2)
	IS*L6*	1
	IS*Sau2*	6
IS*110*	IS*Spn10*	6
	IS*LHe4*	14 (1)
	IS*L4*	1
IS*30*	IS*1139*	16 (3)
	IS*Sag3*	2
	IS*Ljo1*	2 (1)
	IS*1070*	1
IS*4* sub-group IS*Pepr1*	IS*L5*	9
	IS*Lre1*	3 (1)
IS*Lre2*	IS*Lcr2*	11 (4)
IS*4* sub-group IS*4*	IS*1675*	10
IS*66*	IS*Swo2*	4
	IS*Cth11*	4
IS*200*/IS*605* sub-group IS*1341*	IS*Lhe65*	2
	IS*Bth17*	1
IS*1182*	IS*Lac*	3 (1)
IS*200*/IS*605*	IS*Ljo5*	2
IS*L3*	IS*Lhe2*	1
	IS*Sm4*	1
IS*NCY*	IS*H7A*	2

*The number reported in parenthesis refers to the copies of ISs containing a truncated or frame-shifted transposase gene

Figure S1. Representation of Tn*7088* integration and excision. Tn*7088* excises from the *L. crispatus* M247 chromosome producing a circular form and a reconstitution of *att*B insertion site. In the circular form of Tn*7088* the left and right ends are joined by *att*Tn which is identical to *att*L whereas the reconstituted *att*B site is identical to *att*R. *att* sites are represented as filled rectangles, chromosomal genes as arrows, Tn*7088* as a green bar. PCR primers used for circular form and reconstituted *att*B site detection and quantification are depicted as dark and light grey arrowheads, respectively. Frequency of circular form and reconstituted target site is reported in parenthesis. Figure is not scaled.

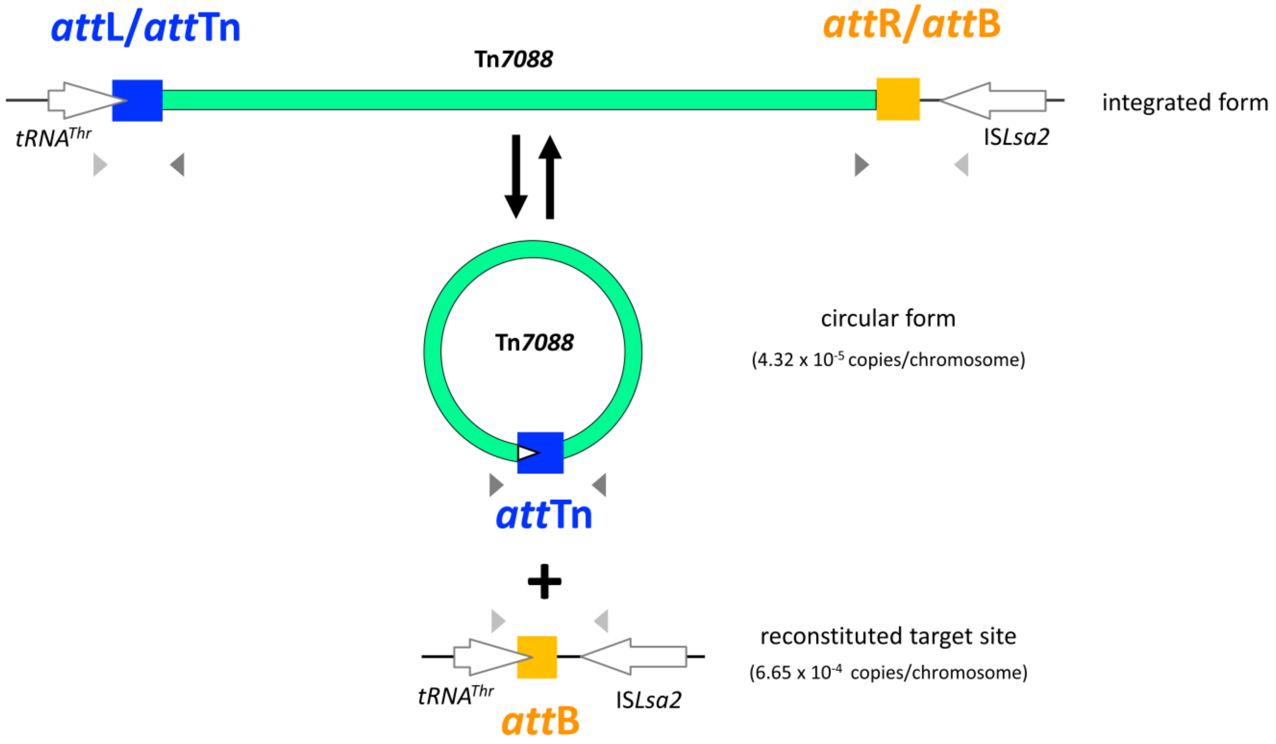

Supplement: Supplementary material 1 [file mgen-9-1150-s001.pdf]
